# The Relation of Physiological Study to Pathology and Therapeutics

**Published:** 1884-03

**Authors:** 


### The Relation of Physiological Study to Pathology and Therapeutics.

In an interesting lecture in *Science*, Dr. H. Newell Martin says on this subject:

I must find time to say a few words as to the connection of physiology with pathology and therapeutics. The business of the physiologist being to gain a thorough knowledge of the properties and functions of every tissue and organ of the body, he has always had for his own purposes to place these tissues under abnormal conditions. To know what a muscle or a gland is, he has to study it, not merely in its normal condition, but when heated or cooled, supplied with oxygen or deprived of it, inflamed or starved, and see how it behaves under the influence of curare, atropine, and other drugs. From the very start of physiological laboratories, a good deal of the work done in them has necessarily been really experimental pathology and experimental therapeutics. I suppose to-day that at least half of the work published from physiological laboratories might be classed under one or other of these heads. And what has been the fruit? I can here refer only to one or two examples. It is not too much to say, that, though inflammation is the commonest and earliest recognized of pathological states, we really knew nothing about it until the experimental researches of Lister, Virchow, and Cohnheim; and that all we really know as to the nature of fever is built on the similar researches of Bernard, Haidenhain, Wood, and others. As to therapeutics, so far as giving doses of medicine is concerned, it, still in its very infancy, had its birth as exact science in physiological laboratories. Every modern text-book on the subject gives an account of the physiological action of each drug. What the future may have in store for us by pursuit of these inquiries it is hard to limit. The work of Bernard—showing that in curare we had a drug that would pick out of the whole body, and act upon, one special set of tissues, the endings of the nerve-fibers in muscle—and the results of subsequent exact experiments as to the precise action of many drugs upon individual organs or tissues, hold out before us a hope that, perhaps, at no very distant day the physician will know exactly, and in detail, what every drug he puts into his patient is going to do in him.

Pathology and therapeutics, while almost essential branches of physiological inquiry, have, nevertheless, their own special aim; and, now that the physiologists have proved that it is possible to study these subjects experimentally, special laboratories for their pursuit are being erected in Germany, France, and England. These laboratories are stocked with physiological instruments, and carry on their work by physiological methods. Those who guide them, and those who work in them, must be trained physiologists; if not, the whole business often degenerates into a mere slicing of tumors and putting up of pickled deformities; pathological anatomy is a very good and important thing in itself, but it is not *pathology*. Looking at the vast field of pathological and therapeutic research open to us, and bearing in mind the certainty of the rich harvest for mankind which will reward those who work on it, I regard as one of my chief duties here to prepare in sound physiological doctrine, and a knowledge of the methods of experiment, students who will afterwards enter laboratories of experimental pathology and pharmacology immediately connected with our medical school.

Memory.—The *Medical Record* very sensibly says: A man's memory is like his stomach. To do its best work it must have good treatment. It must neither be neglected nor overloaded. It can easily be so abused by neglect, or by irregular and unsystematic employment, as to become chiefly a cause of annoyance and discomfort; or, again, it can be so overworked and heavily taxed that it becomes practically the chief organ or agent of the entire system, every other portion dwindling in its comparison. The latter course is the great danger of those who value the help of a tenacious memory. Both memory and stomach are valuable, not in proportion to the burdens they can carry, but in proportion to their training for their part in the work of the system as a whole; and either of them is made effective as much by what is kept from it, as by what is packed into it.

